# Epigenetic priming restores the HLA class-I antigen processing machinery expression in Merkel cell carcinoma

**DOI:** 10.1038/s41598-017-02608-0

**Published:** 2017-05-23

**Authors:** Cathrin Ritter, Kaiji Fan, Annette Paschen, Sine Reker Hardrup, Soldano Ferrone, Paul Nghiem, Selma Ugurel, David Schrama, Jürgen C. Becker

**Affiliations:** 1 Department of Translational Skin Cancer Research, University Hospital Essen, Essen, Germany; 2 German Cancer Consortium (DKTK), Essen, Germany; 3 German Cancer Research Center (DKFZ), Heidelberg, Germany; 4 Department of Dermatology, Medical University of Graz, Graz, Austria; 5 Department of Dermatology, University Hospital Essen, Essen, Germany; 6 Department of Immunology and Vaccinology, Technical University of Denmark, Frederiksberg, Denmark; 7 Department of Surgery, Massachusetts General Hospital, Harvard Medical School, Boston, MA USA; 8 Division of Dermatology, Department of Medicine, University of Washington, Seattle, WA United States; 9 Department of Dermatology, University Hospital Würzburg, Würzburg, Germany

## Abstract

Merkel cell carcinoma (MCC) is a rare and aggressive, yet highly immunogenic skin cancer. The latter is due to its viral or UV-associated carcinogenesis. For tumor progression MCC has to escape the host’s immuno-surveillance, e.g. by loss of HLA class-I expression. Indeed, a reduced HLA class-I expression was observed in MCC tumor tissues and MCC cell lines. This reduced HLA class-I surface expression is caused by an impaired expression of key components of the antigen processing machinery (APM), including LMP2 and LMP7 as well as TAP1 and TAP2. Notably, experimental provisions of HLA class-I binding peptides restored HLA class-I surface expression on MCC cells. Silencing of the HLA class-I APM is due to histone deacetylation as inhibition of histone deacetylases (HDACs) not only induced acetylation of histones in the respective promoter regions but also re-expression of APM components. Thus, HDAC inhibition restored HLA class-I surface expression *in vitro* and in a mouse xenotransplantation model. In contrast to re-induction of HLA class-I by interferons, HDAC inhibitors did not interfere with the expression of immuno-dominant viral proteins. In summary, restoration of HLA class-I expression on MCC cells by epigenetic priming is an attractive approach to enhance therapies boosting adaptive immune responses.

## Introduction

Merkel cell carcinoma (MCC) is a rare neuroendocrine cancer of the skin, but its incidence has tripled over the last 20 years^1, 2^. Based on the disease-specific mortality rate, it is more lethal than melanoma^3^. Nevertheless, spontaneous remissions of both primary MCC as well as metastatic lesions are frequently reported and explained by adaptive immune responses^4, 5^; thus, MCC appears to be a prime candidate for immunotherapy. Indeed, recent clinical trials demonstrated the efficacy of immune checkpoint blocking antibodies^6, 7^. However, at least half of the patients were characterized by a primary resistance to checkpoint blockade, and 14% of the responding patients developed secondary resistance at a median follow-up of 33 weeks. Characterization of the mechanisms underlying immune-resistant cancer progression may contribute to the rational design of strategies to improve the efficacy of immunotherapy in patients suffering from advanced MCC.

The immunogenicity of MCC is based on the association of MCC with a polyomavirus, *i*.*e*. the Merkel cell polyomavirus (MCPyV) in about 80% of cases^8^. MCPyV is integrated into the host cell genome, and tumor cells critically depend on the expression of the virally encoded large and small T antigen (LT and sT, respectively)^9, 10^. We previously reported CD8^+^ T-cell responses targeting LT- and sT-derived epitopes in the majority of MCC patients^11^. Notably, intratumoral infiltration of CD8^+^ T cells is associated with an improved prognosis^12^. Unfortunately, strong intratumoral CD8^+^ T-cell infiltration is a sporadic event in MCC, being present in only 5% of tumors^13^. Lack of T-cell infiltration may reflect different immune escape strategies utilized by MCC cells such as inhibition of cellular immune responses via PD1/PD-L1 signaling^14, 15^, and/or defects in classical HLA class-I antigen expression^16^. Reduction of class-I human leukocyte antigens (HLA) surface expression readily explains MCC’s resistance to PD-1/PD-L1 blockade, as adaptive T-cell responses critically depend on MHC class-I-restricted antigen presentation.

HLA class-I molecules are expressed on the cell surface as heterodimers consisting of the HLA heavy chain and the β_2_-microglobulin (β_2_m) light chain, stabilized by binding of the presented peptide epitope^17^. An intricate network of proteases, peptidases, transporters and chaperone molecules, known as the antigen processing machinery (APM), is necessary to achieve peptide processing, transportation and loading on HLA class-I molecules^18^. Key component of the APM is the proteasome, a multi-protein complex composed of several subunits, including the interferon-inducible LMP2 and LMP7 and the transporter complex which is composed of the subunits TAP1 and TAP2^18^.

APM component expression in cancer cells is down-regulated often by epigenetic mechanisms and - less frequently - by structural mutations. These epigenetic mechanisms includes promoter methylation and/or histone hypoacetylation, and is particularly effective as most APM (*LMP2*, *LMP7*, *TAP1*, *TAP2*) and classical HLA genes (*HLA-A*, -*B* and -*C*) are located in the MHC gene cluster on chromosome 6p^19^. Herein, we demonstrate epigenetic silencing of APM genes in the majority of MCCs *in situ* and *in vitro* resulting in markedly reduced classical HLA class-I expression. HLA class-I expression, however, can be restored in cell lines as well as in a pre-cinical mouse model by pharmacological inhibition of histone deacetylases (HDACs).

## Results

### MCC is characterized by a reduced HLA class-I expression *in situ* and *in vitro*

**Figure 1:**
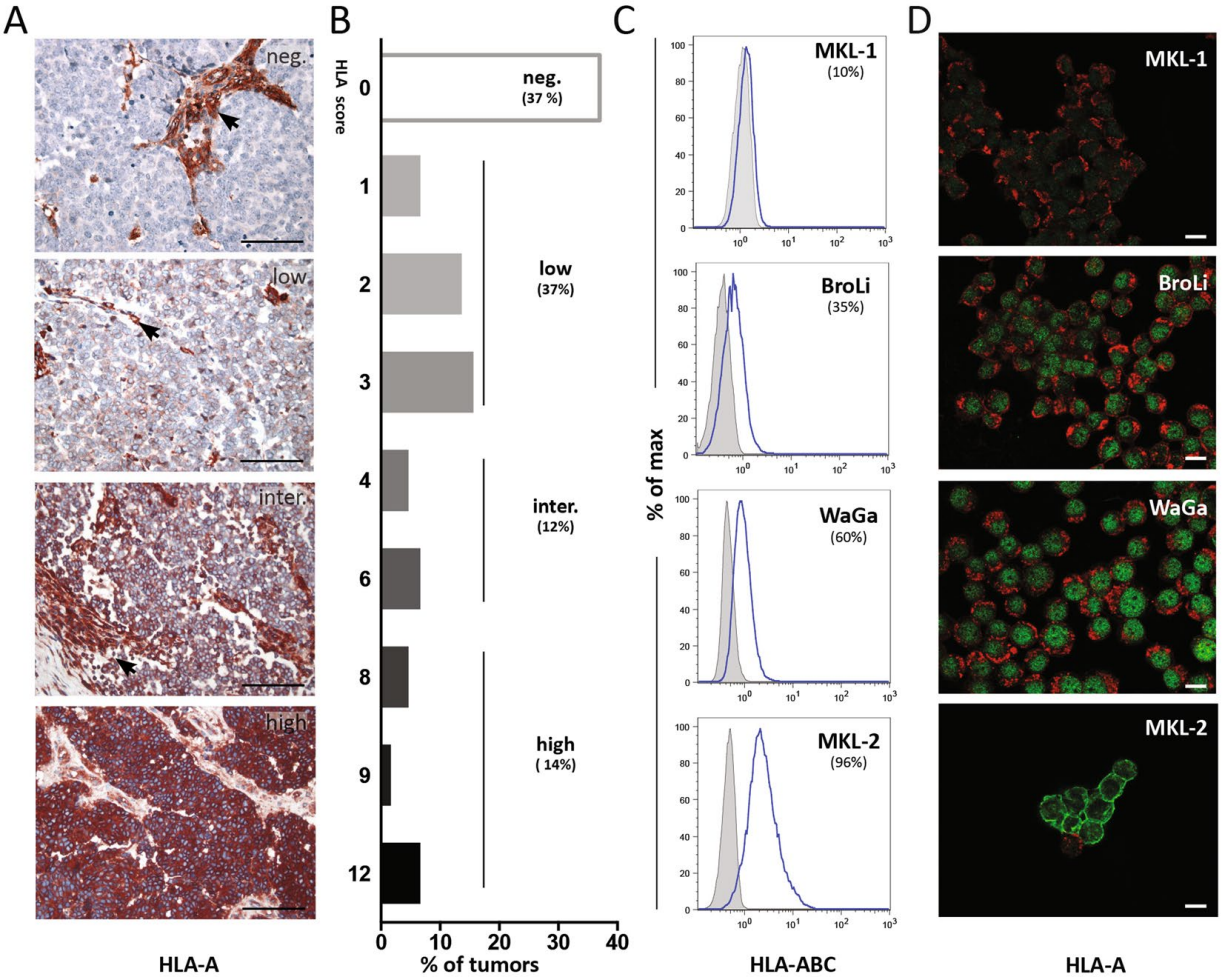
HLA class-I expression by MCC tumors *in situ* and MCC cell lines *in vitro*. (**A**,**B**) 56 MCC tumor samples of 40 patients were analyzed by immunohistochemistry for the expression of HLA-A. Samples were scored from 0 to 3 for staining intensity and from 0 to 4 for the frequency of positive tumor cells; a combined HLA-A expression score was calculated by multiplying both scores. One representative tumor for each HLA score group (negative, low, intermediate and high) is depicted in (**A**); Arrows indicate stromal cells as an internal positive control; scale bar represents 100 µm. (**C**,**D**) Four MCPyV^+^ MCC cell lines were analyzed for their HLA class-I expression *in vitro*. (**C**) HLA class-I surface expression was detected by flow cytometry using an HLA-ABC detecting antibody (clone W6/32, blue line); matched isotype controls are depicted as grey filled histogram. One representative histogram of triplicates is shown for each cell line. (**D**) The cellular localization of HLA class-I was determined by immune fluorescence using wheat germ agglutinin (Alexa fluor 647, red) as membrane marker and a HLA-A detecting antibody (clone EP1395Y) with a dylight 488 labeled secondary antibody (green). Representative images for each cell line were captured with a confocal microscope; scale bars represent 10 µm.

HLA-A expression *in situ* was analyzed by IHC in 56 MCC lesions from 40 patients using an HLA-A specific antibody (clone EP1395Y; Fig. 1A). A HLA-A staining score was compiled as described in the material and method section (Fig. 1B). In line with Paulson *et al*.*’s* observations, 37% (n = 20) MCC lesions entirely lacked HLA-A expression (HLA score 0), 37% (n = 21) were characterized by a low expression (HLA score 1–3), 12% (n = 7) by an intermediate expression (HLA score 4–6), whereas only 14% (*n* = *8*) expressed high amounts of HLA-A molecules on most of the tumor cells (HLA score 8–12). Next, we analyzed a series of eight MCC cell lines for their HLA class-I surface expression with an HLA-A, -B and -C detecting antibody (clone W6/32) by flow cytometry revealing differential expression levels of HLA class-I (supplementary Fig. S1). Four MCC cell lines representing almost no (MKL-1), low (BroLi), intermediate (WaGa) or high (MKL-2) HLA class-I surface expression were selected for further analysis (Fig. 1C). The subcellular localization of HLA-A was determined with an HLA-A detecting antibody (clone EP1395Y) by immunofluorescence (Fig. 1D), confirming the respective HLA class-I surface expression determined by flow cytometry for each cell line. Immunofluorescence also captured that despite reduced HLA class-I surface expression of WaGa, BroLi, and MKL-1, HLA-A is still present in their cytoplasm (Fig. 1D).

### Reduced HLA class-I cell surface expression on MCC cells is associated with HLA class-I APM component down-regulation

**Figure 2:**
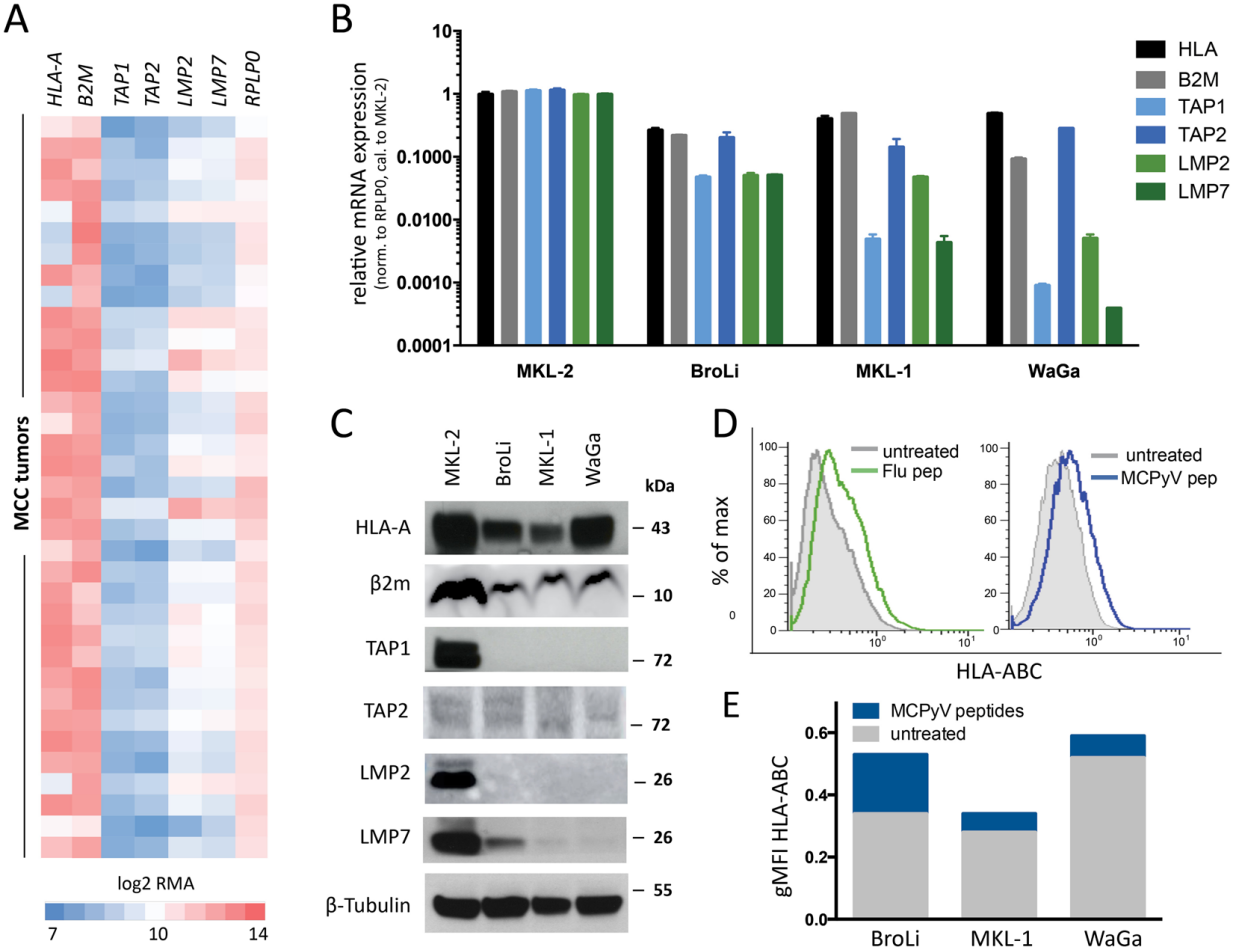
Reduced HLA class-I expression in MCC is associated with an impaired antigen processing machinery (APM). (**A**) RMA normalized expression values of gene expression array GSE22396, were obtained from the GEO database. RMA values were log2 transformed and are depicted as heat map with expression values ranging from 7 (blue = low expression) to 14 (red = high expression). *HLA-A*, *B2M*, *TAP1*, *TAP2*, *LMP2* and *LMP7* mRNA expression is shown in comparison to RPLP0. (**B**) mRNA expression of *HLA-A* (*black*), *B2M* (*grey*), *TAP1* (*light blue*), *TAP2* (*dark blue*), *LMP2* (*light green*) and *LMP7* (*dark green*) in 4 MCC cell lines was determined by RT-qPCR in triplicates using specific primers; C_T_ values were normalized to *RPLP0* and calibrated to a set of ΔC_T_s of MKL-2; relative mRNA expression is depicted as mean + SEM. (**C**) Protein expression in 4 MCC cell lines was determined by immunoblot of whole cell lysates using antibodies specific for HLA-A, β_2_m, TAP1, TAP2, LMP2 and LMP7; β-tubulin served as loading control. (**D**,**E**) MCC cell lines with low (BroLi, MKL-1) and intermediate (WaGa) HLA class-I surface expression were incubated with saturating amounts (10 µM) of a flu peptide mix or MCPyV encoded large and small T antigen and VP1 derived epitopes binding with high affinity to the respective HLA-A molecules or an irrelevant peptide cocktail for at least 24 h (WaGa and MKL-1) or 48 h (BroLi). HLA class-I surface expression was determined by flow cytometry using an HLA-ABC detecting antibody. Induction of HLA class-I surface expression after stabilization with a flu peptide mix (green line), exemplarily shown for MKL-1, or the specific MCPyV peptide mix, exemplarily depicted for BroLi (blue line). An irrelevant peptide cocktail (grey filled) was used as control (**D**). Comparison of HLA class-I surface expression is depicted as geometric mean fluorescence intensity (gMFI) after incubation with MCPyV derived high affinity (blue) or an irrelevant peptide control (grey) for all analyzed MCC cell lines (**E**).

To elucidate the mechanism(s) leading to the reduced HLA class-I expression in MCC, we analyzed gene expression data from 35 cryopreserved MCC tumors, accessible at the gene expression omnibus (GEO) database (GSE22396), for *HLA-A* and *B2M*. Surprisingly, only ~25% of tumors (*n* = *8*) expressed low or intermediate levels of *HLA-A* mRNA, whereas the majority (~75%, *n* = *27*) contained high amounts of *HLA-A* specific mRNA (Fig. 2A). *B2M* mRNA was also expressed at intermediate to high levels in the majorities of tumors. This discrepancy between *HLA-A* heavy chain mRNA and HLA-A membrane expression could be due to a lack of MHC complex stabilization by bound peptides. Thus, we next analyzed the mRNA expression of the HLA class-I APM components, *TAP1*, *TAP2*, *LMP2* and *LMP7* in the same data set (GSE22396). To this end, *TAP1* and *TAP2* mRNAs were expressed at very low levels in all analyzed tumors, and *LMP2* and *LMP7* mRNAs at low to intermediate levels in ~75% of tumors (*n* = *27*) (Fig. 2A). To determine whether this expression indeed reflect transcript levels in MCC cells and not cells of the tumor microenvironment, we analyzed the four MCC cell lines with varying degrees of HLA-A surface expression for gene expression of *HLA-A*, *B2M*, and the APM components. In line with the results generated from the analysis of their expression in tumor tissue, *HLA-A* and *B2M* mRNAs were present at high levels in all MCC cell lines (Fig. 2B; supplementary Fig. S2), irrespective of the MHC class-I membrane expression (Fig. 1C and D). However, the three MCC cell lines with reduced MHC class-I membrane expression (BroLi, MKL-1 and WaGa) were characterized by lowered *LMP2*, *LMP7*, *TAP1 and TAP2*, mRNA levels (Fig. 2B). Only MKL-2, a cell line showing high MHC class-I expression, contained high levels of most of the APM- component specific mRNAs (Fig. 2B, supplementary Fig. S2). It should be noted, that *TAP2* mRNA expression was also low in MKL-2 cells. To confirm this observation at the protein level, we performed immunoblots of total cell lysates with HLA-A-, β_2_m-, TAP1-, TAP2-, LMP2- and LMP7-specific mAbs (Fig. 2C), revealing that HLA-A and β_2_m were expressed in all analyzed MCC cell lines, while TAP1 and LMP2, LMP7 expression was largely restricted to the MKL-2 cell line (Fig. 2C). In line with the mRNA expression, TAP2 protein was only sparsely expressed in all the analyzed cell lines (Fig. 2C).

### External supply of MCPyV-derived peptide epitopes stabilizes HLA class-I surface expression by MCC cells

Most MCCs analyzed by us expressed high levels of both *HLA* heavy and *B2M* light chain mRNA whereas the respective expression of APM components was low. Since empty β_2_m-HLA class-I heavy chain complexes are rapidly removed from the cell surface and subsequently degraded^20, 21^, reduced HLA class-I surface expression by MCC cells is likely to be due to deficient antigen processing. Indeed, addition of either a pool of HLA class-I-restricted T-cell epitopes derived from influenza virus or MCPyV increased the HLA class-I surface expression of MKL-1, BroLi and WaGa cell lines (Fig. 2D,E). The used MCPyV peptides have been described before^11^ and were selected according to the HLA-A type of the tested MCC cell line (Supplementary Table S3). In cells with deficient antigen processing such as the TAP-deficient T2 cell line, an excess of externally provided peptides binding to the respective HLA molecules stabilizes HLA class-I complexes in an APM-independent manner^22^. Thus, reduced HLA class-I cell surface expression in MCC is at least in part due to a diminished availability of peptides for HLA class-I stabilization.

### Transcription of APM genes in MCC cell lines is epigenetically silenced by histone hypoacetylation

Expression of APM genes can be silenced by DNA methylation and/or histone hypoacetylation at the *MHC* gene cluster^19^. Analyses of GSE22396 revealed that a high abundance of *histone deacetylase* (*HDAC*) mRNAs (sum of *HDAC 1*–*10*) was negatively correlated with the mRNAs encoding LMP2, LMP7, TAP1 and TAP2, (R squared = 0.32, p = 0.0006; supplementary Fig. S3A). Since no such correlation was observed for methyltransferases (supplementary Fig. S3B), and treatment of MCC cell lines even with high doses (5 µM) of two distinct methyltransferase inhibitors (5-azacytidine and RG108) had no effect on HLA class-I surface expression (supplementary Fig. S3C), we focused on the relevance of histone hypoacetylation.

**Figure 3:**
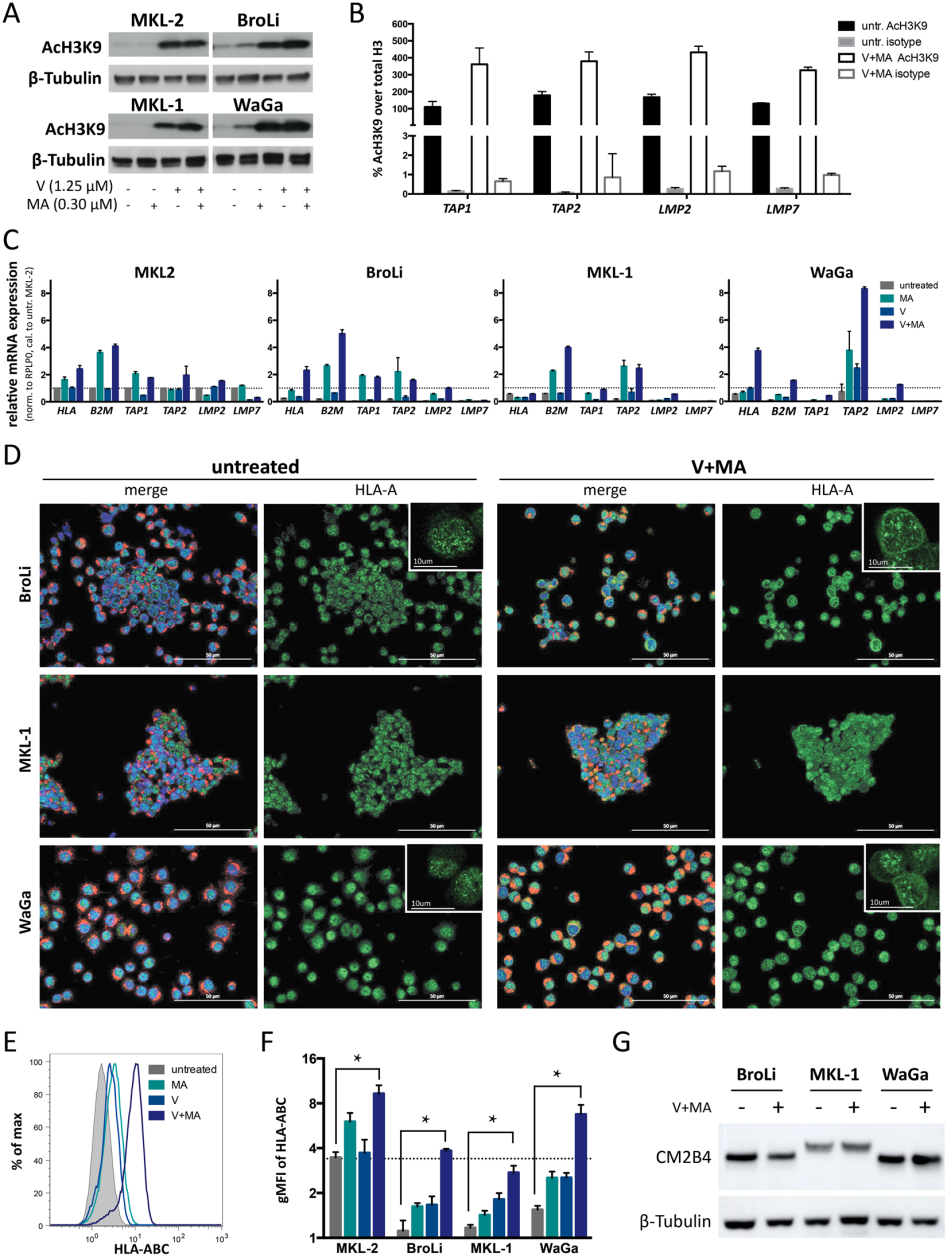
Reduced expression of antigen processing machinery genes is mediated by histone hypoacetylation and is increased by pharmacologic histone deactylase inhibition. For all experiments the indicated MCC cell lines were analyzed without treatment (grey) and after treatment with mithramycin A (MA, turquoise), vorinostat (V, light blue), or the combination thereof (V + MA, dark blue). (**A**) Global H3K9 acetylation of untreated, V, MA or V + MA treated MCC cell lines was determined by immunoblot with an AcH3K9 antibody; β-tubulin served as loading control. (**B**) Chromatin immunoprecipitation (ChIP) assay was performed with untreated or V + MA treated WaGa cells followed by a qRT-PCR using *TAP1*, *TAP2*, *LMP2* or *LMP7* promoter specific primers. C_T_ values of AcH3K9 antibody or rabbit IgG isotype precipitated DNA were normalized to histone H3 antibody precipitated DNA as described in material and methods. Experiments were performed in triplicates twice and results are expressed as mean + SEM. (**C**) mRNA expression of *HLA-A*, *B2M*, *TAP1*, *TAP2*, *LMP2* and *LMP7* was determined by RT-qPCR in triplicates; C_T_ values were normalized to *RPLP0* and calibrated to the ΔC_T_ value of untreated MKL-2 cells; relative mRNA expression is depicted + SEM for MKL-2, BroLi, MKL-1 and WaGa. (**D**) HLA class-I intracellular localization was determined via immunofluorescent staining using an HLA-A detecting antibody (clone EP1395Y) with a dylight 488 labeled secondary antibody (green). Wheat germ agglutinin (Alexa fluor 647, red) was used to stain cellular membranes and DAPI (blue) served as nuclear stain. Single cell images in the upper right corner of the overview image were acquired using confocal microscopy. (**E**,**F**) HLA class-I cell surface expression was determined by flow cytometry using a HLA-ABC specific antibody as exemplified for WaGa (**E**); the results for all cell lines analyzed are depicted as the geometric mean fluorescence intensity (gMFI) of HLA class-I (HLA-ABC) staining, + SEM in three independent experiments (**F**). Statistical analysis was performed using the Friedman test as indicated. (**G**) Viral protein expression was determined by immunoblot of whole cell lysates using an antibody specific for LT (clone CM2B4) depicting the different truncated LTs characteristically for MCC cells. β-tubulin served as loading control.

Global histone acetylation of MKL-2, WaGa, MKL-1 and BroLi was determined before and after HDAC inhibition by immunoblot (Fig. 3A). As the culture of these MCC cell lines for 24 hours in clinically relevant concentrations of vorinostat (V), i.e. the plasma level of 1.25 µM observed under current treatment regimens^23^, resulted only in a modest induction of global histone acetylation, we combined V with mithramycin A (MA). MA is a clinically approved DNA-binding cytotoxic antibiotic synergizing with HDAC inhibitors by (i) transcriptionally inhibiting the compensatory induction of certain HDACs^24^ and (ii) by preventing the formation of SP1/HDAC inhibitory complexes at the promoters’ GC box^25^. Notably, the combined treatment of MCC cell lines with V and MA resulted in a marked induction of global histone acetylation. Next, histone H3K9 promoter acetylation of *LMP2*, *LMP7*, *TAP1 and TAP2* was analyzed in untreated or V plus MA treated WaGa cells by chromatin immune precipitation (ChIP). Treatment with V plus MA increased histone H3K9 acetylation in all analyzed promoter regions (Fig. 3B). mRNA expression of APM components *LMP2*, *LMP7*, *TAP1 and TAP2* was measured by RT-qPCR before and after HDAC inhibition in MKL-2, WaGa, MKL-1 and BroLi cells (Fig. 3C). The combined treatment with V and MA strongly induced mRNA expression of the analyzed APM component encoding genes in all the tested MCC cell lines. As suggested by the increased HLA class-I membrane expression in response to the external supply of HLA-binding peptides, HDAC inhibition-induced expression of APM components resulted in an increased HLA class-I surface expression as demonstrated by immunofluorescence (Fig. 3D) and flow cytometry (Fig. 3E and F). Confocal microscopy also visualized, that HDAC inhibition resulted in a cytoplasmic conglomeration of HLA-A protein, most likely in cellular compartments where MHC-class-I complexes are assembled, loaded and transported to the cell surface.

Surprisingly, MA treatment alone had a stronger effect on APM gene expression than V alone. However, only HDAC inhibition by V plus MA had a marked synergistic effect on HLA class-I surface expression (Fig. 3E and F). It is important to note, that the combined treatment did not interfere with the expression of MCPyV-encoded proteins, which represent the source of immunogenic epitopes in MCPyV^+^ MCC (Fig. 3G).

### HDAC inhibition induces histone acetylation and HLA class-I stabilization *in vivo*

**Figure 4:**
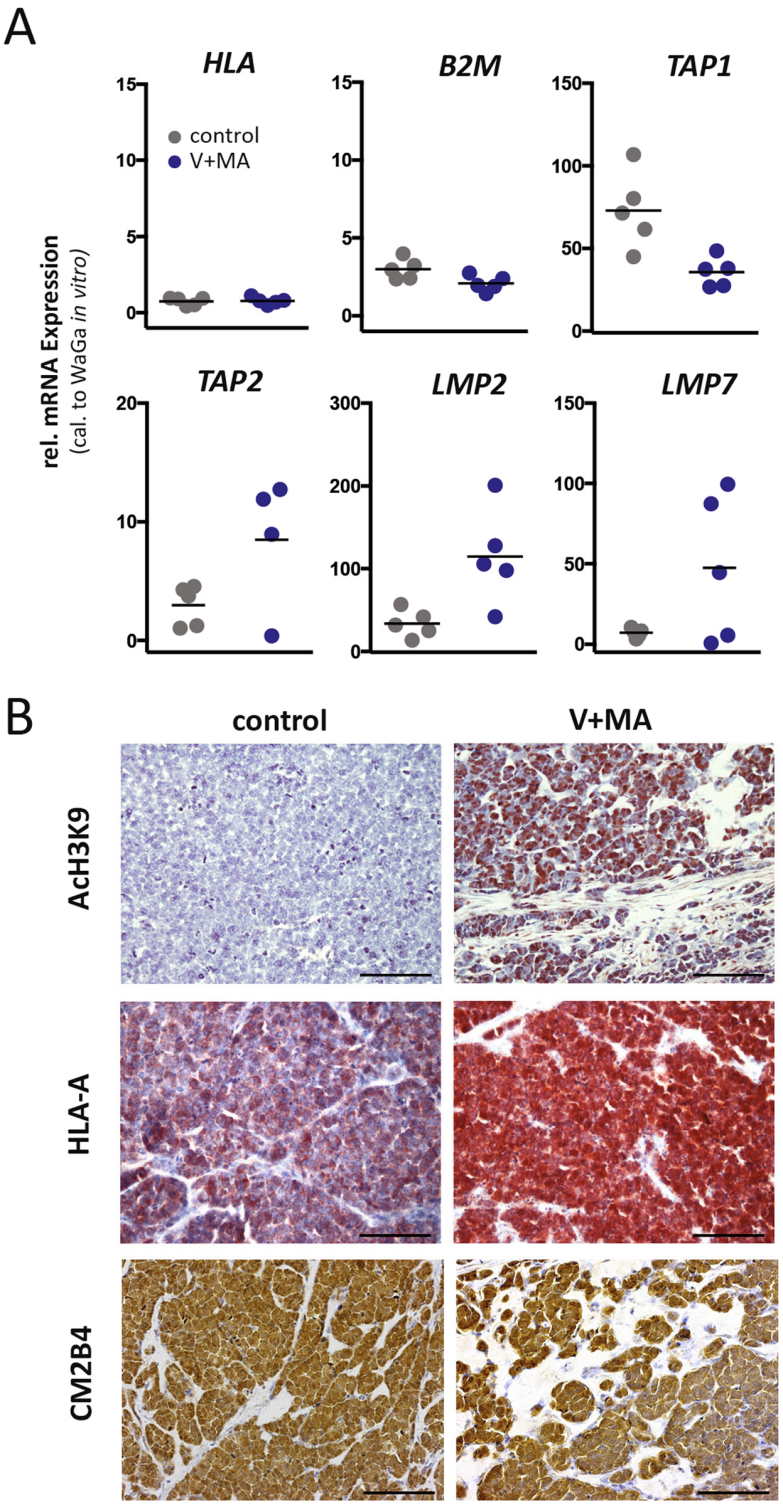
Histone deactylase inhibition induces histone H3K9 acetylation and HLA class-I expression and does not reduce LT expression *in vivo*. NOD.CB17/*Prkdc* ^*scid*^ mice (*n* = *5* for each treatment group) bearing xenotransplanted WaGa tumors were treated with the drug carrier polyethylene glycol (control, grey), or the combination of vorinostat and mithramycin A (V + MA, dark blue) as described in materials and methods. (**A**) mRNA was isolated from cryopreserved tumors and RT-qPCR was performed using primers for *HLA-A*, *B2M*, *TAP1*, *TAP2*, *LMP2* and *LMP7*. C_T_ values were normalized to *RPLP0* and calibrated to *in vitro* cultured WaGa cells. (**B**) Immunohistochemistry was performed on sections of FFPE fixed tumors using antibodies specific against AcH3K9, LT (clone CM2B4) and HLA-A. A representative example for each group is depicted. Scale bars represent 100 µm.

Next, we translated these observations into a MCC xenotransplantation model based on WaGa cells and NOD/SCID mice^26^. Once the induced tumors reached a volume of approximately 100 mm^3^, treatment with i.p. injections of either V plus MA or the respective drug carriers was started. Mice were treated for two weeks at concentrations equivalent to those applied in humans. Forty-eight hours after the last dosage, animals were sacrificed and tumors harvested. In analogy to the *in vitro* results, treatment of mice with V plus MA significantly increased the transcription of TAP2, *LMP2 and LMP7* in xenotransplanted MCC tumors (p < 0.05; Fig. 4A). However, the expression of *TAP1 in vivo* was not increased by this treatment; this finding may reflect the strong *TAP1* induction by xenotransplantation itself. This induction may be due to the fact that NOD/SCID mice are comprised of functional B and T cells, but still harbor functional dendritic cells and NK cells expressing interferon gamma^27^. Immunohistochemistry of the xenotransplants confirmed that these effects were indeed due to HDAC inhibition, as histone H3K9 acetylation was strongly induced by treatment of mice with V plus MA (Fig. 4B). Consequently, the combined treatment enhanced the HLA class-I surface expression on WaGa xenotransplants (Fig. 4B). In accordance to the *in vitro* results RT-qPCR did not demonstrate any increase in *HLA-A* and β_*2*_ *m* mRNA expression in response to treatment with V plus MA, supporting the assumption that HDAC inhibitor mediated HLA class-I induction on MCC cells is largely mediated by an enhanced antigen processing (Fig. 4A). Furthermore, it is important to note that MCPyV large T antigen (LT) protein expression was not altered in MCC xenotransplants by V plus MA treatment (Fig. 4B).

## Discussion

Virus-associated malignancies like MCC are highly immunogenic and are consequently subjected to a strong immuno-selective pressure. Thus, these cancers can only progress if efficient immune escape strategies are operative. Indeed, although most patients harbor MCPyV-derived epitope-specific CD8^+^ T cells circulating in the blood^11, 28^, and that in most MCC cases the oncogenic phenotype critically depends on continuous expression of the MCPyV early genes^9, 10^, brisk T-cell infiltrates in MCC tumors are rare^13, 29^.

Consequently, immune modulation is a promising therapeutic option for MCC patients, which is currently tested in several clinical trials including: autologous MCPyV-specific CD8^+^ T-cell transfer, checkpoint blocking antibodies, or cytokine-based therapies using tumor stroma-targeting antibody-IL2 fusion proteins and *in vivo* electroporation of IL-2-coding plasmids (reviewed in ref. 30). Notably, very promising results on the efficacy of immune checkpoint blocking antibodies have already been reported^6, 7^. These therapeutic approaches depend on MCC-specific adaptive immune responses. For cytotoxic T cells a cognate recognition, i.e. interaction of the T-cell receptor with suiTab. peptide/HLA class-I complexes presented on the surface of the target cells, is essential. Thus, it is not surprising that diminished HLA class-I expression on cancer cells is one of the major immune escape mechanisms both spontaneously and in response to immunotherapy^31–34^. The molecular mechanisms to achieve such an HLA class-I loss are multiple and vary depending both on the type of tumor as well as on the individual patient^35^.

In line with recent reports, we demonstrate here that the impaired HLA class-I expression is a frequent immune escape mechanism of MCC, which however, appears not to be genetically fixed as it can be therapeutically overcome^16^. Actually, we demonstrate here that the common underlying molecular mechanism of HLA class-I loss observed in the MCC cell lines investigated by us is epigenetic silencing of the antigen presentation machinery (APM): The transporter subunits TAP1 and TAP2 as well as the immunoproteasome subunits LMP2 and LMP7 are simultaneously down-regulated by histone hypoacetylation. While genetic aberrations in single components of the APM have been reported^18^, the synchronized down-regulation of several APM components appears to be an even more efficient immune escape mechanism^36^.

Viral infection of cells is frequently associated with an inflammatory response. Thus, infected cells are exposed to cytokines such as interferon gamma, which results in the replacement of certain catalytic subunits of the proteasome by LMP2 and LMP7 to allow the formation of the immunoproteasome^37^. The immunoproteasome not only generates a broader spectrum of peptides for presentation by HLA class-I complexes, but is also much more efficient in processing viral proteins, which are poorly processed by the regular proteasome^38, 39^. Thus, epigenetic silencing of LMP2 and LMP7 is a particularly suiTab. means of immune evasion for cancers with a viral carcinogenesis such as MCC. Indeed, viruses can change the host epigenome in order to escape immune responses (reviewed in ref. 40). In this regard, viral infection can alter the host epigenome directly via the interaction of viral components with the epigenetic machinery of the infected cell^41, 42^, or indirectly due to the increased immune selective pressure caused by the expression of viral antigens. It has to be elucidated which scenario is operative in MCC.

In any case, this highly effective immune escape mechanism may be reversed pharmacologically: Epigenetically silenced genes of the HLA class-I locus are effectively re-induced by HDAC inhibition^43–45^. To this end, treatment of MCC cells with the HDAC inhibitor vorinostat in combination with the Sp1 inhibitor mithramycin A resulted in the transcription of the APM components followed by HLA class-I surface expression. Notably, we observed this effect at clinically relevant concentrations and could translate these findings into a pre-clinical *in vivo* model. Consequently, epigenetic immunological priming could be readily used to overcome primary or secondary resistance to PD-1/PD-L1 blocking antibodies^6, 7^. Indeed, the combination of different HDAC inhibitors with immune checkpoint blocking antibodies is currently tested in solid tumors, including melanoma and non-small cell lung cancer (Supplementary Table S4). This notion is particularly attractive for MCC, since HDAC inhibition also induces the expression of activating and co-activating molecules such as NKG2D ligands^46^. An alternative means to up-regulate HLA class-I surface expression are interferons. We have previously reported that both type I and II interferons are potent inducers of HLA class-I surface expression in MCC^11, 16^. However, interferons reduce the expression of the MCPyV encoded sT and LT transforming early genes^26^, i.e. the source which most of the immuno-dominant T cell epitopes are derived from^11^. In line with this, treatment of two MCC patients with MCPyV positive primary tumors and metastasis with interferon alpha had no therapeutic effect^47^.

We here demonstrate that epigenetic priming by HDAC inhibition appears to be an effective means to improve viral antigen processing and surface presentation in the majority of MCCs. MCC patients characterized by primary or secondary resistance to immunotherapy are likely to be rendered responsive by HDAC inhibition. As demonstrated here, testing for HLA class-I expression together with the H3K9 acetylation status of tumor lesions by immunohistochemistry, which can be readily performed in FFPE samples, may prove to be a valuable predictive biomarker of response to immunotherapy^48–50^. As mentioned above, there are currently several clinical trials ongoing in which the combination of HDAC inhibition and immunotherapy is tested as treatment of solid tumors (Supplementary Table S4); based on the presented data, MCC should be one of these cancers.

## Material and Methods

### MCC tumor samples

A total of 56 formalin-fixed and paraffin-embedded (FFPE) tumor samples from 40 unselected MCC patients presenting at the Department of Dermatology, Medical University of Graz, were used for analysis. All tumor samples were excised for diagnostic or therapeutic reasons between 1992 and 2013, and were confirmed to be Merkel cell carcinoma according to established histological and immunohistochemical diagnostic guidelines. FFPE specimens included primary lesions, recurrent tumors, as well as metastases to the skin or lymph nodes. All studies on human material were approved by the institutional review board of the Medical University of Graz (24–295 ex 11/12). Informed consent has been obtained and the methods were carried out in accordance with the approved guidelines.

### Immunohistochemistry

Immunohistochemistry (IHC) was performed on FFPE tissue as describe before^47^. In short antigens were retrieved using a citrate buffer (Retrieval solution, cat. no. S1699, pH 6, Dako, Glostrup, Denmark) in a steamer at 100 °C for 30 minutes. Antibodies used in this study are listed in Supplementary Table S1. Samples were incubated for 1 hour with an HLA-A specific antibody (clone EP1395Y, Abcam, Bristol, UK) diluted 1:1000 in antibody diluent (Dako). After incubation with a biotinylated secondary antibody and addition of streptavidin peroxidase, the detection was obtained using ImmPACT NovaRED Peroxidase Substrate (Vector Laboratories, Burlingame, CA, USA). Three independent investigators (CR, DS, JCB) classified the staining intensity on a scale from zero to three and the percentage of HLA-A positive cells as follows: 0% = 0, 1–25% = 1, 26–50% = 2, 51–75% = 3, >75% = 4. The HLA-A staining score was calculated by multiplying the score for staining intensity with the percentage of positive cells (min = 0, max = 12).

### Cell culture

The classical MCPyV^+^ MCC cell lines BroLi, MKL-1, MKL-2, and WaGa have been described before^51^. All cell lines were authenticated by STR analysis on regular bases (last performed in June 2016). Cell lines were maintained in RPMI-1640 (PAN Biotech, Aidenbach, Germany) supplemented with 10% fetal bovine serum (FBS; Biochrom, Berlin, Germany) and 1% penicillin/streptomycin (Biochrome, Berlin, Germany). For treatment with specific inhibitors, cells were cultured at a concentration of 1 × 10^6^ cells/ml in 6-well plates. Inhibitors were dissolved according to the manufacturers’ guidelines and used at 1.25 µM for vorinostat (Selleckchem, Munich, Germany), 0.3 µM for mithramycin A (Sigma, St. Louis, MO, USA), 5 µM for RG108 and 5 µM 5-azacytidine (Selleckchem) for 24 h if not otherwise stated.

### Flow cytometry

Cell surface expression of HLA class-I was determined by flow cytometry. 1 × 10^6^ cells were incubated with an FITC conjugated anti-HLA-ABC antibody (Supplementary Table S1, clone W6/32, BioLegend, San Diego, CA, USA) in PBS supplemented with 0.1% bovine serum albumin (BSA) for 90 minutes at 4 °C in the dark. After washing, cells were stained with 10 µg/ml 7-aminoactinomycin (7AAD, Sigma) for viable/non-viable discrimination, and measured on a FC500 Flow Cytometer (Beckman Coulter, Brea, CA, USA). Data were analyzed with FlowJo Version 8.7 software (TreeStar, Sunnyvale, CA, USA).

### Immunofluorescence

Before immunofluorescence staining, MCC cells were attached to coverslips by poly-L-lysine coating. In short, coverslips were coated with 0.01% of poly-L-lysin (Sigma) for 10 minutes, and placed after one wash with PBS in a 12-well plate. 1 ml of cell suspension was added per well and incubated for 30 minutes at 37 °C in 5% CO_2_. After excess media was removed, the cells were washed with PBS once and incubated with 5 µg/ml wheat germ agglutinin (WGA, conjugated with Alexa Fluor 647) for 30 minutes at room temperature to stain cellular membranes. After three 5 minute washing steps with PBS, cells were fixed with ice cold acetone for 10 minutes, followed by three more washes with PBS and blocking with 1% bovine serum albumin (BSA) in PBS for one hour. Samples then were incubated with an antibody specific for HLA-A (Supplementary Table S1, clone EP1395Y, Abcam) diluted 1:250 in blocking solution over night at 4 °C. After three washes with PBS a dylight 488 conjugated goat-anti-rabbit IgG (Thermo Scientific, Waltham, MA, USA) was added at a 1:200 dilution in blocking reagent for 1 hour at room temperature. Cells were washed three more times and nuclei were stained with DAPI. Cells were washed with PBS three more times and embedded with ProLong Dimond Antifade Mountant (Invitrogen, Carlsbad, CA, USA) and incubated at room temperature in the dark for 24 h before imaging. For spheroid imaging a Zeiss Axio Observer.Z1 with Apotome.2 system was used. Subcellular imaging of individual cells was performed with a Leica SP5 inverse confocal laser scanning microscope.

### Quantitative real time polymerase chain reactions

mRNA of *in vitro* propagated cells or cryopreserved xenografts was isolated, transcribed into cDNA and RT-qPCR was performed as described before^46^. *RPLP0* served as endogenous control using specific primers and Taqman probe, listed in Supplementary Table S2. The mRNA expression of *HLA-A*, *B2M*, *TAP1*, *TAP2*, *LMP2*, *LMP7* was detected using SYBR green assays with specific primers listed in Supplementary Table S2. Relative quantification was calculated by the ΔΔCt method.

### Immunoblot

Cell lysates were generated by exposing 3 × 10^6^ cells to protein extraction buffer supplemented with a proteinase inhibitor cocktail as described before^52^. Lysates were subjected to sodium dodecyl sulfate polyacrylamide gel electrophoresis (SDS-Page), subsequently transferred to a nitrocellulose membrane (Bio-Rad, Hercules, CA, USA), incubated first for 1 hour in blocking buffer, and then overnight at 4 °C with the respective primary antibodies (Supplementary Table S1) in predetermined dilutions in phosphate buffered saline (PBS) supplemented with 0.1% Tween 20 (PBST): anti-HLA-A (clone EP1395Y, Abcam, 1:4000), anti-β-Tubulin (clone TUB2.1, Sigma, 1:200), anti-B2M (1:500), anti-TAP1(1:500), anti-TAP2(1:500), anti-LMP2 (1:500) and anti-LMP7 (1:500); antibodies against B2M, TAP1, TAP2, LMP2 and LMP7, that are not commercially available have been described before^53^. Global histone acetylation was determined using an anti-acetyl-H3K9 (AcH3K9) antibody (Cell Signaling, Cambridge, UK, clone C5B11, 1:500). MCPyV LT antigen was detected using the antibody clone CM2B4 (Santa Cruz, Dallas, TX, USA, 1:250). The next day, membranes were washed and incubated for 1 hour with the appropriate peroxidase-coupled secondary antibodies (Dako), followed by visualization using the ECL Western Blotting Substrate (Pierce, Waltham, MA, USA).

### HLA class-I stabilization assay

MCC cell lines were incubated with 10 µM of a flu-peptide-pool (PANATecs, Heilbronn, Germany) or a peptide mixture of MCPyV LT-, sT- and VP1-derived epitopes known to match the respective HLA types of the used cell lines (Supplementary Table S3)^11^. The flu-peptide-pool served as a well established positive control for HLA class-I stabilization^54^. As negative control, an irrelevant peptide with no HLA binding motif was used. Incubation was performed until optimal HLA class-I stabilization was observed, i.e. 24 hours for WaGa and MKL-1 and 48 hours for BroLi. Quantification of HLA class-I expression by flow cytometry was performed as described above.

### Chromatin immune precipitation (ChIP)

ChIP assays were performed using the SimpleChiP® Enzymatic Chromatin IP Kit with agarose beads (Cell Signaling). In brief, proteins were cross-linked to DNA with 1.5% formaldehyde for 10 minutes. Nuclear membranes were broken up using the Bioruptor sonicator (Diagenode, Seraing, Belgium) set to high, for 2 cycles of 10 minutes (30 seconds on/30 seconds off). Afterwards, antibodies against histone H3 (clone D2B12, Cell Signaling), acetyl-histone H3 (Lys9) (AcH3K9) (clone C5B11, Cell Signaling), or normal rabbit IgG (cat. no. 2729, Cell Signaling) were used for immune precipitation. The precipitated DNA was analyzed by qRT-PCR using primers specific for the *TAP1*, *TAP2*, *LMP2* (*PSMB9*) or *LMP7* (*PSMB8*) promoter region; primer sequences are given in Supplementary Table S2. The percentage of acetylated histones H3 (AcH3K9) was normalized to total histones H3 and calculated using following equation:

$$ \% {\rm{AcH}}3\,{\rm{over}}\,{\rm{total}}\,{\rm{H}}3=100\ast 2({{\rm{C}}}_{{\rm{T}}}{\rm{H}}3-{{\rm{C}}}_{{\rm{T}}}{\rm{AcH}}3{\rm{K}}9).$$

### Xenotransplantation experiments

Six-week-old female NOD.CB17/Prkdc^scid^ mice were obtained from Charles River Laboratories (Erkrath, Germany) and housed under specific pathogen-free conditions. Tumors were induced by s.c. injection as described before^26^. Twenty-four days after tumor cell inoculation at a volume of approximately 100 mm^3^ treatment was started. Mice were divided into four groups of five ensuring an equal overall tumor burden. Mice were treated with 60 mg/kg vorinostat in combination with 0.2 mg/kg bodyweight or the drug carrier 45% Polyethylene glycol (PEG-400, Sigma) intra-peritoneal injection as describe befor^46^. Mice were treated five consecutive days per week for two weeks. IHC was performed as described in the Immunohistochemistry section. Anti-acetyl-histone H3 (Lys9) (AcH3K9) (cell signaling, cat. no. 9671) was diluted 1:50 and the LT specific antibody clone CM2B4 was diluted 1:200 (Supplementary Table S1). All animal studies were approved by the Austrian ministry of education and science according to the regulations for animal experimentation (BMWF-66.010/0151-II/3b/2012).

### Statistics

Statistical analyses were performed using Graphpad Prism 6.0 Software (Graphpad Software Inc., San Diego, CA, USA). Cell culture experiments were analyzed using Friedman test, a paired non-parametric ANOVA. A p-value smaller than 0.05 was considered significant and indicated by * in the figures.

## Electronic supplementary material

Supplementary DOC File
