# Supplementary material for: Epigenetic priming restores the HLA class-I antigen processing machinery expression in Merkel cell carcinoma
Source: Sci Rep. 2017 May 23;7:2290. doi: 10.1038/s41598-017-02608-0 (PMC5442125; doi:10.1038/s41598-017-02608-0)
Supplement: Supplementary file 1 — Supplementary DOC File [file 41598_2017_2608_MOESM1_ESM.doc]

**Supplementary Information to:**

**Epigenetic priming restores the HLA class-I antigen processing machinery expression in Merkel cell carcinoma**

Cathrin Ritter, Kaiji Fan, Annette Paschen, Sine Reker Hardrup, Soldano Ferrone, Paul Nghiem, Selma Ugurel, David Schrama, Jürgen C. Becker

**Table S1**: Antibodies used in this study

| **Clone** | **Specificity** | **Supplier/ Reference** |
| --- | --- | --- |
| **EP1395Y** | HLA-A | Abcam |
| **W6/32** | HLA-ABC | BioLegend |
| **B2M** | β_2_m | 22 |
| **SY1** | LMP2 | 22 |
| **HB-2** | LMP7 | 22 |
| **NOB-1** | TAP1 | 22 |
| **NOB-2** | TAP2 | 22 |
| **C5B11** | AcH3K9 | Cell Signaling |
| **D2B12** | Hisone H3 | Cell Signaling |
| **CM2B4** | MCPyV LTA | Santa Cruz |
| **Tub2.1** | β-tubulin | Sigma |

**Table S2: Primer sequences**

|  | **forward** | **reverse** |
| --- | --- | --- |
| **HLA-A** | GCGGCTACTACAACCAGAGC | GATGTAATCCTTGCCGTCGT |
| **B2M** | TCTCTGCTGGATGACGTGAG | TAGCTGTGCTCGCGCTACT |
| **TAP1** | TCAGGGCTTTCGTACAGGAG | TCCGGAAACCGTGTGTACTT |
| **TAP2** | ACTGCATCCTGGATCTCCC | TCGACTCACCCTCCTTTCTC |
| **LMP2 (PSMB9)** | TCAAACACTCGGTTCACCAC | GGAGAAGTCCACACCGGG |
| **LMP7 (PSMB8)** | CATGGGCCATCTCAATCTG | TCTCCAGAGCTCGCTTTACC |
| **RPLP0** | CCATCAGCACCACAGCCTTA | GGCGACCTGGAAGTCCAACT |
| **RPLP0 probe** | ATCTGCTGCATCTGCTTGGAGCCC | |
| **TAP1 promoter** | TGGGTTCTGGGAAATGTGGAG | TGGCCCAAAGAATCAAGACC |
| **TAP2 promoter** | ATGAGCACCGGTTACTCAGG | ATGAGAAATCATGGGGGTGGAG |
| **LMP2 promoter** | GGAAAGCGAAATCGAAAGCG | TGATTTCCACGCTTGCTACC |
| **LMP7 promoter** | ACCTCTTACTGTAACCCATCGC | AATGATGGGTCAAGGGTCTTCC |

**Table S3: Peptide sequences**

| **Cell line** | **HLA-A type** | **LT peptide** | **sT peptide** | **VP1 peptide** |
| --- | --- | --- | --- | --- |
| **BroLi** | A11/A2* | ASFTSTPPK | IMMELNTLWSK | ASVPKLLVK |
| **MKL-1** | A3 | ASFTSTPPK | IMMELNTLWSK | KMALHGLPR |
| **WaGa** | A1/A2 | P**V**IMMELN**T**L | KTLEETDYCLL | ALHGLPRYFNV |

**Table S4: Clinical trials combining HDACis and immunotherapy**

| **NCT number** | **HDACi** | **Immunotherapy** | **Cancer** | **Phase** |
| --- | --- | --- | --- | --- |
| **NCT02032810** | panobinostat | ipilimumab | Melanoma | 1 |
| **NCT02437136** | entinostat | pembrolizumab | NSCL, Melanoma | 1/2 |
| **NCT02697630** | entinostat | pembrolizumab | Uveal Melanoma | 2 |
| **NCT02909452** | entinostat | pembrolizumab | advanced solid tumors | 1 |
| **NCT02619253** | vorinostat | pembrolizumab | renal and urothelial cell carcinoma | 1/2 |
| **NCT02453620** | entinostat | Ipilimumab, nivolumab | Breast | 1 |
| **NCT02915523** | entinostat | Ipilimumab,  avelumab | Ovarian | 1/2 |
| **NCT02805660** | mocetinostat | durvalumab | advanced solid tumors, NSCLC | 1/2 |

HDACi: HDAC inhibitor; NSCLC: non small cell lung cancer, ipilimumab: anti-CTLA-4 antibody; pembrolizumab: anit-PD-1 antibody; nivolumab: anti-PD-1 antibody; avelumab: anti-PD-L1 antibody; durvalumab: anti-PD-L1 antibody

**Supplementary figures**

**Fig. S1: HLA class-I expression on the surface of MCC cell lines.**

HLA class-I surface expression of MCC cell lines WaGa, MKL-1, MKL-2, LoKe, AlDo, BroLi, WoWe and PeTa was detected by flow cytometry using an HLA-ABC detecting antibody (clone W6/32, blue line); isotype controls are depicted as grey filled histogram.

**
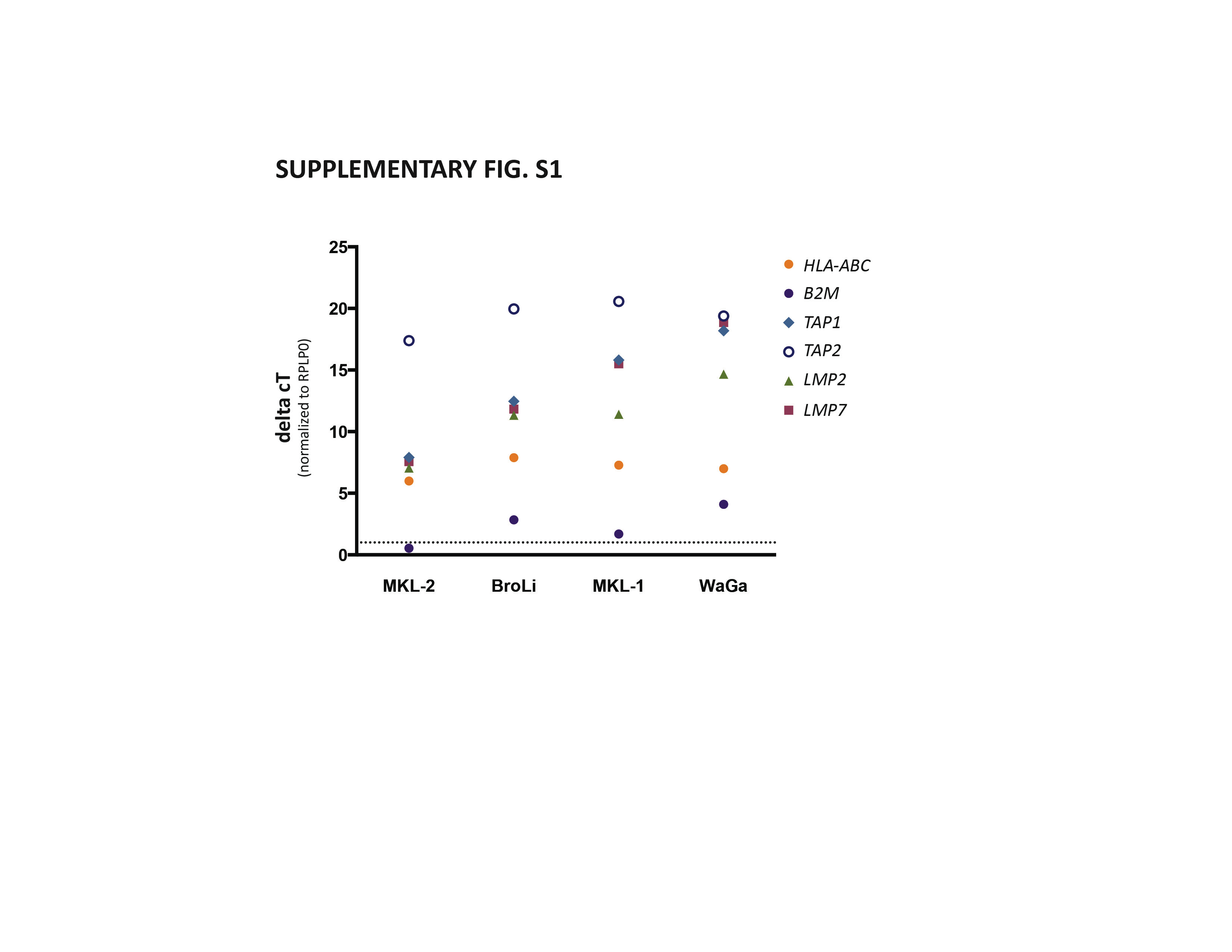
**

**Fig. S2: *HLA-A* and *B2M* mRNA is expressed in high abundance, whereas antigen processing machinery (APM) genes are variably expressed in MCC cell lines.**

*HLA-A* (yellow dot), *B2M* (purple dot), *TAP1* (blue diamond), *TAP2* (blue circle), *LMP2* (green triangle) and *LMP7* (red square) mRNA expression in the MCC cell lines MKL-2, BroLi, MKL-1 and WaGa was determined by qRT-PCR using specific primer sets. Depicted are ΔC_T_ values (C_T; target gene_ - C_T; RPLP0_), which correlate inversely with expression.

**
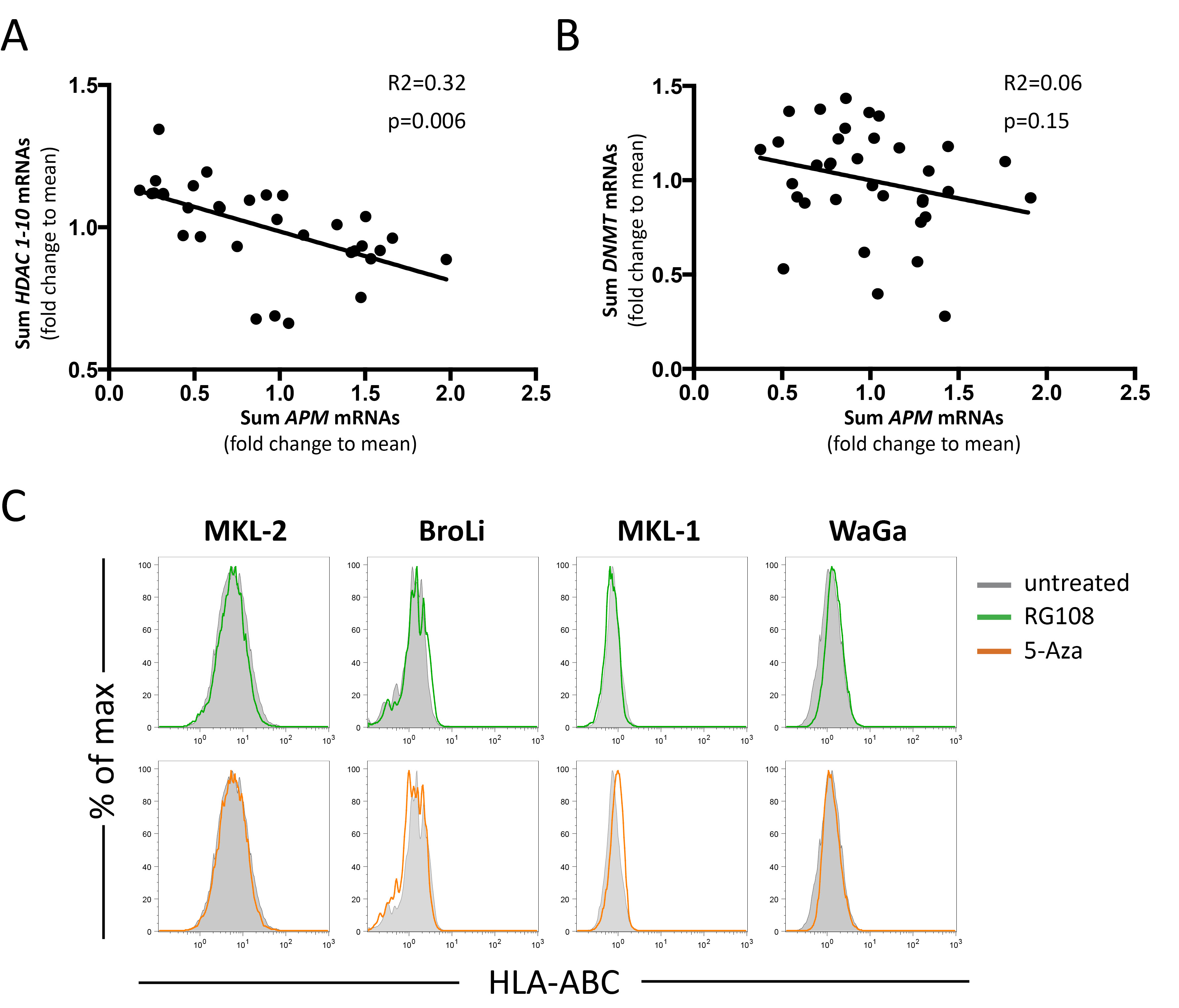
**

**Fig. S3: Histone deacetylase (HDAC) but not DNA methyltransferase (DNMT) mRNA expression is inversely correlated with expression of antigen processing machinery (APM) genes.**

RMA normalized mRNA expression values of class I and class II HDACs (*HDAC1-10*) **(A)** or DNMTs (1/3) **(B)** genes and of the antigen processing machinery (APM), i.e. *TAP1*, *TAP2*, *LMP2*, *LMP7,* were extracted from a gene expression array in the GEO database (GSE22396). Fold change expression to mean was calculated for each individual sample and gene. The sum of relative expression of *APM* genes was correlated with the sum of genes of *HDACs* (**A**, R^2^=0.32, p=0.0006.) or DNMTs (**B**, R^2^ =0.06, p=0.15). **(C)** MCC cell lines were left untreated (grey filled) or treated with 5 µM of the DNMT inhibitors RG108 (green line) or 5-azacytidine (orange line) for 24 hours. HLA class-I surface expression was determined by flow cytometry using an HLA-ABC specific antibody (clone W6/32).
